# Multi-Component Analysis of *Ilex Kudingcha* C. J. Tseng by a Single Marker Quantification Method and Chemometric Discrimination of HPLC Fingerprints

**DOI:** 10.3390/molecules23040854

**Published:** 2018-04-09

**Authors:** Huan Yi, Jie Zhou, Xueying Shang, Zhongxiang Zhao, Qian Peng, Mingjuan Zhu, Chenchen Zhu, Chaozhan Lin, Qide Liu, Qiongfeng Liao, Lei Zhang

**Affiliations:** School of Pharmaceutical Sciences, Guangzhou University of Traditional Chinese Medicine, Guangzhou 510006, China; yi317296805@163.com (H.Y.); zhoujie_gz@163.com (J.Z.); 13430286922@163.com (X.S.); zzx37@163.com (Z.Z.); 18394812810@163.com (Q.P.); zhumingjuan123@163.com (M.Z.); zhuchenchen@vip.sina.com (C.Z.); linchaozhan@sina.com (C.L.); liuqd020@126.com (Q.L.); liaoqf2075@yahoo.com (Q.L.)

**Keywords:** *Ilex kudingcha* C. J. Tseng, quantitative analysis of multi-components with a single marker (QAMS), chemomerics, relative correction factor, HPLC

## Abstract

The quantitative analysis of multiple components with a single marker (QAMS) method was firstly established for simultaneous determination of 18 active components in *Ilex kudingcha* C. J. Tseng by HPLC. Using rutin, isochlorogenic acid A and kudinoside A as internal refererence substances (IRS), compatibility results showed that the relative correction factors (RCFs) of all compounds showed good reproducibility under different chromatographic conditions. On the basis of previous studies, the accuracy of the QAMS method was systematically evaluated by investigating the influences of curve intercept, analytes and IRS concentration. The results showed that the concentration (especially at low level) of analytes and curve intercept were the major influencing parameters for the LRG-QAMS method (LRG = linear regression), whereas the influence of IRS concentration seemed more apparent in terms of the AVG-QAMS method (AVG = average). The two approaches were complementary with each other. In addition, hierarchical clustering analysis (HCA), principal components analysis (PCA) and similarity analysis (SA) were performed to differentiate and classify the samples based on the contents of 18 marker compounds. The results of the different chemometric analyses were completely consistent with each other, and could be supported by the quantification results.

## 1. Introduction

*Ilex kudingcha* C. J. Tseng (Kudingcha in Chinese), the dried leaves of the genus *Ilex* in the family Aquifoliaceae, is widely distributed in the Hainan, Hubei, Guangdong, Guangxi and Hunan provinces of China. It has been used as an ethnomedicine for the treatment of diseases relative to dyslipidosis for millennia [1]. Nowadays it has become a hotspot in traditional Chinese medicine (TCM) research due to its significant effects on cardiovascular-related diseases and metabolic syndrome [2,3]. *Ilex kudingcha* C. J. Tseng is commonly applied as a strategy in the clinic for adjuvant therapy of diabetes, hypertension, obesity, and hyperlipidemia mainly based on its antioxidant and immunoregulatory properties [4], and has also been developed into various health care products to improve patient compliance.

Pharmacologically active ingredients in *Ilex kudingcha* C. J. Tseng have been elucidated to be phenolic acids, triterpenoids and flavonoids, which have activities against inflammatory processes, viruses, hypertension, atherosclerosis, mutations, hyperglycemia and cancer [5]. It is generally recognized that it is the multiple components contained in the TCMs, rather than any single or several compounds, which act on multiple targets or interact with different biochemical pathways to synergistically perform the overall therapeutic functions [6]. Therefore, the three types of the dominant constituents are usually considered as “marker compounds” for the chemical evaluation or standardization of *Ilex kudingcha* C. J. Tseng [7]. Unfortunately, most of these quantification methods have been limited to the determination of mono- and dicaffeoylquinic acids, flavonoids (rutin, kaempferol) and common triterpenes (ursolic acid, oleanolic acid) [7]. To date, there are few reports concentrating on the quantification of multiple characteristic triterpenes in *Ilex kudingcha* C. J. Tseng [8,9]. Those reported quantification methods have the following drawbacks: (1) the numbers of analytes are very limited; (2) the widespread application of these methods is limited due to difficult access to standards of the most unique triterpenes. Obviously, the quality standards of *Ilex kudingcha* C. J. Tseng are still far from sufficient for an integrated quality control strategy [7,10], and it is imperative to develop more effective and comprehensive analytical methods to address this problem.

Quantitative analysis of multi-components by a singer marker (QAMS) is an economic, convenient and environmentally friendly method for the simultaneous assay of multiple components, in which only one reference standard will be needed to determine all the analytes in the sample when the method is officially accepted, and thus resolves the bottleneck problem of reference substance scarcity [11]. Since the method was firstly proposed by Wang et al. in China for quality analysis of Akebiae Caulis, it has received more attention in the pharmacopoeia of China, Europe, India and America, and is even applied for the quantification study of 20 kinds of herbals by the American herbal pharmacopoeia [12]. However, the QAMS method is mainly aimed at constituents which have similar molecular structures, UV spectral and chromatographic behaviors [12,13]. However, as the three types of the dominant constituents in *Ilex kudingcha* C. J. Tseng have quite different structural features and properties, a QAMS method using three internal reference standards (IRS) for each type of constituents was developed to facilitate the simultaneous assay of eighteen constituents in *Ilex kudingcha* C. J. Tseng using a multi-wavelength monitoring technique. Quantification of so many analytes by the HPLC-DAD technique is more difficult than mere determination of important trace constituents among thousands of chemicals. Meanwhile, integration of these analytes with wide range of polarities into one analysis procedure also brings more significant obstacles to this study.

In addition to QAMS, fingerprinting has also highly been recommended by academia as a powerful approach for quality control of medicinal herbs and herbal products [14]. Chemical pattern recognition is acknowledged as a more objective and effective method for identifying a particular herb from related species and evaluating the similarities and differences among medicinal materials in comparison with determination of single or multiple markers. Chemometric analysis methods, such as similarity analysis (SA), hierarchical clustering analysis (HCA) and principal components analysis (PCA) are widely used for chemical classification and chromatographic profile aligning. In this study, eighteen components including eight phenolic acids, seven triterpenes and one flavonoid were simultaneously assayed by using three common IRS, and detected at three different wavelengths. The QAMS method was evaluated by comparing the calculated results with those obtained from validated traditional external standard method [15], and HCA, PCA and SA was adopted to discriminate 15 batches of *Ilex kudingcha* C. J. Tseng collected from different locations in China based on the quantitative results.

## 2. Results

Referring to the chromatograms of HPLC-DAD detection, all chromatographic peaks could be divided into three groups by their corresponding maximum absorption wavelength, that was 210 nm for K1, K2, K3, K4 and K5, 260 nm for R1, R2, R3, R4, R5 and R6, and 326 nm for C1, C2, C3, C4, C5, C6 and C7. For the eighteen components, rutin (R4, λ_max_ = 260 nm), isochlorogenic acid A (C6, λ_max_ = 326 nm) and kudinoside A (K3, λ_max_ = 210 nm) with, easily accessibility and good chemical stability properties were selected as the IRS for the three types of constituents.

### 2.1. Validation of the Traditional External Standard Method

**Figure 1:**
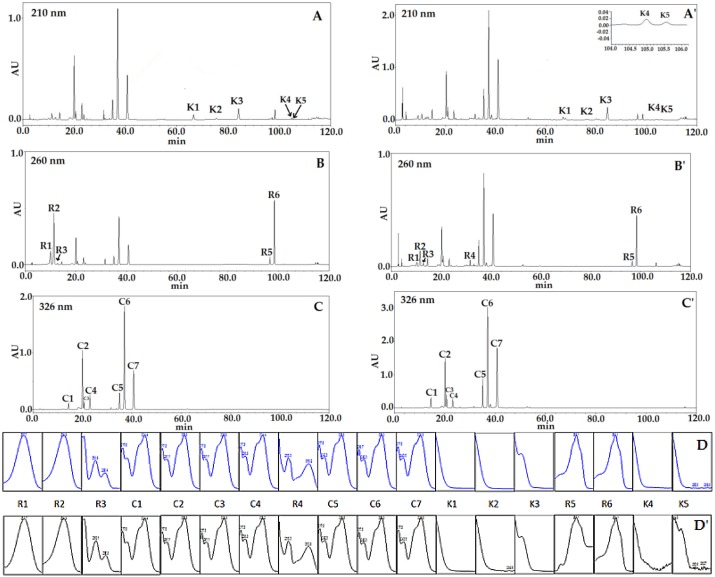
HPLC chromatograms of eighteen mixed reference standards (**A**–**C**); sample solution (No. 13) at different wavelength (**A’**–**C’**); and UV spectra of standards (**D**) and analytes in sample solution (**D’**).

The method specificity was assessed by comparing the consistency of the retention time and UV spectrum of each analyte between a sample and corresponding reference standard. Figure 1 shows a typical separation of a standard mixture (A)–(C) and *Ilex kudingcha* C. J. Tseng extracts (A)’–(C)’ under the optimized chromatographic conditions. All the markers were not only well resolved from background peaks but a good resolution of adjacent peaks within the analysis time was also attained. Figure 1(A–C) and (A’C’) illustrate that the eighteen peaks in the chromatogram of *Ilex kudingcha* C. J. Tseng could all be identified by the corresponding standards. The peak purity detection function of photo-diode array (PDA) detector was used, which confirmed the acceptable purity of the eighteen analytes’ peaks in the samples.

### 2.2. Calibration Curves and Evaluation of RCFs Versus Concentration

A total of 20 μL of different concentration levels (*n* = 6) of mixed standards solution were injected into HPLC systems for the construction of calibration curves. The standard curves for the 18 components showed good linearity with correlation coefficients higher than 0.9990 within the test ranges (Table 1). Meanwhile, RCFs and relative retention time (RT_R_) were calculated based on the linearity data and summarized. The relative standard deviation (RSD) values of RCFs and RT_R_ were less than 4.77% and 0.26%, respectively, which suggested that RCFs and RT_R_ obtained on the same instrument at different concentration level were highly repeatable.

### 2.3. Precision, Repeatability, Stability and Accuracy

The precision was examined from five consecutive injections of the sample placed in the autosampler (10 °C). The repeatability was tested by injecting six independently prepared samples, which were prepared according to the method outlined in the “Preparation of solutions” section. The stability was tested with a sample solution that was stored at room temperature (25 ± 5 °C) and analyzed at 0, 2, 4, 6, 8 and 12 h. The accuracy was determined by a recovery test performed by spiking all the reference standards at middle concentration level (*n* = 6) into a sample (0.1 g) of *Ilex kudingcha* C. J. Tseng powder, followed by extraction and analysis as described in “Preparation of solutions” section. The recovery for each analyte was calculated based on the equation of recovery (%) = 100 × (amount found − original amount)/amount spiked. The results are displayed in Table 2.

### 2.4. Robustness Test of QAMS

#### 2.4.1. Effects of different columns and instruments

ACCHROM C_18_ (4.6 mm × 250 mm, 5 μm), Waters Symmetry C_18_ (4.6 mm × 250 mm, 5 μm) and Phenomenex Synergi Hydro-RP C_18_ (4.6 mm × 250 mm, 5 μm) columns were used to study the effects of different columns. The experiments on different instruments were carried out in different labs, using LC-20A, Agilent 1260 and Waters Alliance E2695 instruments. The results (Table 3) showed that the RCFs and RT_R_ from different columns had good reproducibility with RSD < 4.86% and RSD < 4.94%, respectively, while HPLC instruments had a slight influence on RCFs and RT_R_, with RSD < 5.17% and RSD < 4.92%, respectively.

#### 2.4.2. Effects of different column temperature and flow rate on RCFs

The Shimadzu LC-20A system with a Phenomenex synergi C_18_ column was used to investigate the effects of different column temperatures (25, 28, 30, 32 and 35 °C) and flow rates (0.8, 0.9 and 1.0 mL·min^−1^) on RCFs. The results (Table 4) proved that baseline separation of all marker components would be realized when the column temperature was lower than 30 °C and flow rate less than 1.0 mL·min^−1^. RCFs and RT_R_ kept constant within the temperature range of 25 °C~30 °C, with RSD of 1.14%~6.24% and 0.54%~4.59%, respectively. Three flow rates were examined, and good repeatability of RCF and RT_R_ for the target compounds was obtained with RSDs ranging from 1.45%~5.31% and 0.09%~3.40% respectively.

### 2.5. Sample Analysis

**Figure 2:**
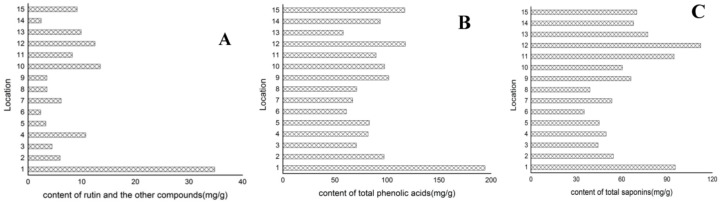
The content of rutin and the other two compounds (**A**); total phenolic acid (**B**) and total saponins (**C**) in 15 batches of *Ilex kudingcha* C. J. Tseng.

The content of 18 compounds was calculated according to the calibration curves, and those that were scattered in the vicinity of the lowest concentration point on the standard curve, were determined with a one point external standard method. The results (Table 5) illustrated that there were remarkable differences among the content of the 18 analytes in different *Ilex kudingcha* C. J. Tseng samples, which could be attributed to the variations of genetics, plant origins, environmental factors, drying process, storage conditions and so on. The respective summation of individual component content grouped by ingredient categories was performed, and the results (Figure 2) showed that the differences of total content for each type components in *Ilex kudingcha* C. J. Tseng samples were less than five times for most herbal samples (except sample No. 1). 

Additionally, we found that the content summation of C2, C6 and C7 accounted for so large proportion of the total phenolic acids (>75%) that the three compounds could be used as index components for quality assessment of *Ilex kudingcha* C. J. Tseng, and the same conclusion could be drawn for K1, K2 and K3 with more than 80% proportion of the total saponins.

### 2.6. Evaluation the Accuracy and Investigation of Influencing Parameters on QAMS Method

**Figure 3:**
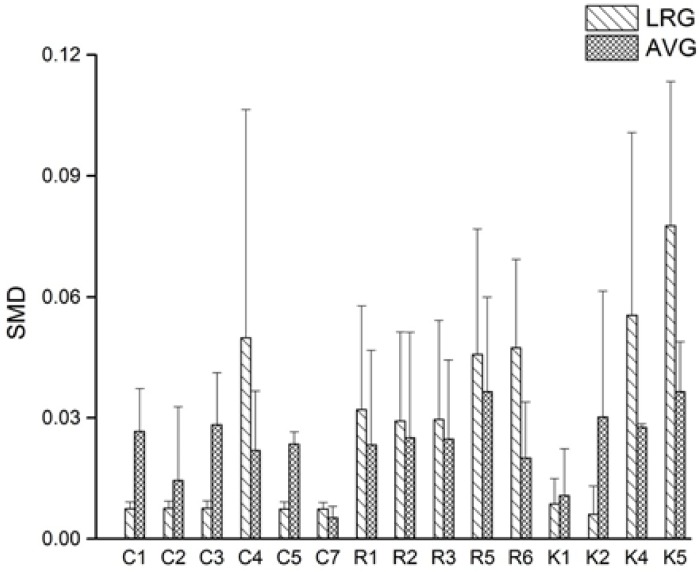
Standard method difference (SMD) of the LRG and AVG method.

To systematically investigate the accuracy of LRG-QAMS and AVG-QAMS method, the two QAMS methods were compared with an external standard method (ES) [15], respectively, by standard method difference (SMD) calculated according to the following equation: SMD% = 100 × (C_ES_ − C_QAMS_)/C_ES_ (7), where C_ES_ and C_QAMS_ represent the concentration of an analyte assayed by the ES method and QAMS method, respectively. As shown in Figure 3 and Table 6, Table 7 and Table 8, a total of 675 SMD datapoints calculated from fifteen quantitative markers in 15 batches of *Ilex kudingcha* C. J. Tseng samples were obtained. The results showed that the two QAMS methods could obtain accordant values compared with the ES method for the assay of the different *Ilex kudingcha* C. J. Tseng samples, but different from previous studies [5,16], the reported deduction that LRG-QAMS method had higher accuracy in comparison with AVG-QAMS method did not seem universal judging from Figure 3.

The influences of two parameters on the accuracy of LRG-QAMS and AVG-QAMS method were investigated, including the content of each quantitative component in the plant materials and the content of IRS. SMDs (total of 675 data) were divided into two groups according to the criteria described as follows: (1) contents of IRS at high, middle and low level; (2) contents of analytes at high, middle and low level.

**Figure 4:**
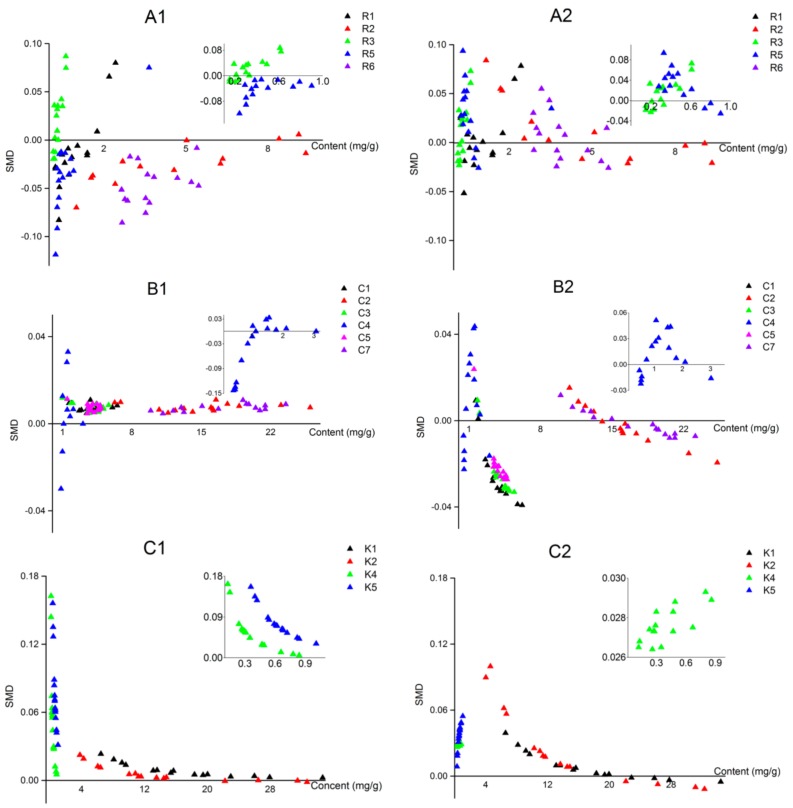
The scatter diagrams that reflect the relationship between standard method differences (SMDs) of fifteen analytes in 15 batches of *Ilex kudingcha* C. J. Tseng assayed by using LRG-QAMS (**A1**~**C1**)/AVG-QAMS (**A2**~**C2**) method and the content of the 15 analytes (X axis represents the content of the 15 analytes, A: R1~R6 except R4, B: C1~C7 except C6, C: K1~K5 except C3; R4, C6 and K3 as the internal standard).

**Figure 5:**
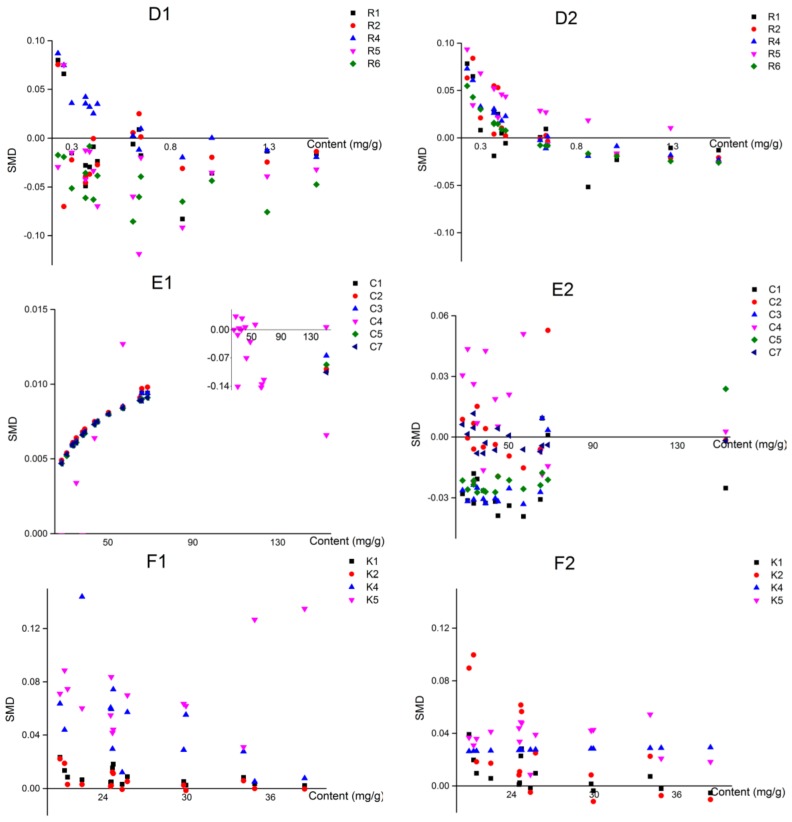
The scatter diagrams reflecting the standard method differences (SMDs) of fifteen analytes in 15 batches of *Ilex kudingcha* C. J. Tseng assayed by using LRG-QAMS (**D1**~**F1**)/AVG-QAMS (**D2**~**F2**) method and the content of the internal referring substances (X axis represents the content of internal referring substances of R4, C6 and K3 in D, E and F scattered gram, respectively).

Scatter diagrams (Figure 4 and Figure 5) were used to investigate the mathematical relationship between these potential influence parameters and SMDs. As shown in the scatter diagrams, the results implied that the accumulation of the component in herbal material rather than that of IRS was the main correlation factor associated with the accuracy of the LRG-QAMS method.

In the case of the AVG-QAMS method, on the contrary, the IRS content seemed a little more predominant impact factor on the accuracy, and SMDs of most components exhibited a decreasing trend with increasing IRS accumulations. However, the influence of the analytes’ content on SMDs did not show a consistent and regularly tendency in the AVG-QAMS method.

### 2.7. Analysis of the Samples

**Figure 6:**
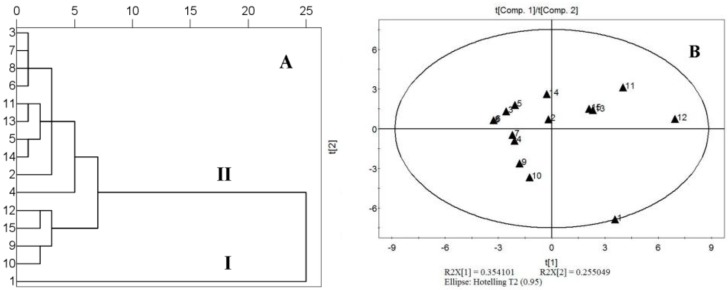
Results of hierarchical clustering analysis (**A**); and principal components analysis (**B**) of the 15 samples of *Ilex kudingcha* C. J. Tseng.

In the HCA analysis, ‘Euclidean distance’ was selected as measurement, and the method of ‘Within-group Linkage’ was applied. As shown in Figure 6A, the fifteen tested populations were classified into two main clusters (I and II) according to their contents. No.1 sample from Hainan with characteristics of significant content differences in each type of components was clearly distinguished from other origin herbal samples. Regarding the similarity calculation, Euclidean distance, correlation coefficient and cosine were adopted as measurements to reflect the similarity of samples (summarized in Table 9). Compared with the Euclidean distance, the measurements of cosine and correlation which ignore the quantitative discrepancy of variables mainly provide qualitative information for TCMs authentication. No. 1 sample, having significant differences in each category component content, gained relatively high score value (>0.94) for cosine and correlation analysis, which proved it was collected from the same genus, and coincident with the results of pharmacognosy identification. On the other hand, the measurement of Euclidean distance also focuses on reflecting the quantitative discrepancy of characteristic variates. The relatively lower score value (approximately 0.90) in the No. 1 sample for Euclidean distance analysis implied that there was a significant discrepancy in the content of marker compounds between No. 1 and other herbal samples. In addition, the determination results of 15 samples were further analyzed and classified by PCA. The two principle components (PC1 and PC2) with more than 60.9% of the whole variances were extracted for analysis. As shown in Figure 6B, the scatter plot was noticeable that No. 1 sample was clearly discriminated from the group, and scattered on the boundary of the circle. The results of HCA, PCA and similarity analysis were exactly consistent with each other.

## 3. Discussion

According to the literature, sonication with methanol was a preferred method. The extraction duration and times were tested. Finally ultrasonic extraction with 100 fold excess of methanol for 30 min was chosen because all the analytes could not only be efficiently extracted, but also well resolved from background peaks.

In order to generate the RCFs of analytes at different wavelengths, it is essential to choose three suitable IRS. The selected IRS should be an index composition meeting four requirements as follows: (1) abundant in sample; (2) stable; (3) easily accessible; (4) having a maximum UV absorption. Meanwhile, a good separation under the chromatographic conditions should be achieved.

The validated traditional external standard method (ES) and QAMS method were employed to assay 15 batches of representative *Ilex kudingcha* C. J. Tseng samples from different locations, which were based on the principle of the linear relationship between a detector response and the levels of components within certain concentration ranges.

As it shown in the results of evaluation the accuracy of QAMS, we found that the SMDs calculated from LRG-QAMS method for those components, the content of which in *Ilex kudingcha* C. J. Tseng samples scattered near the lowest concentration point of the standard curve, suffered relatively more significant deviation from ES results compared with the AVG-QAMS method. Such deviation at low concentration level would be dramatically reduced with the decreasing of intercept values. Only if the intercept value was small enough to be ignored (relative to the product of slope and concentration), the content assayed by LRG-QAMS method would be much closer to ES method calculation. Due to the significant content variability in different source herbal materials, the standard curve usually has to be constructed with a broad liner range. Thus, SMDs values calculated from LRG-QAMS method for the components, content of which is out of or near the lowest concentration level of the standard curve, are remarkable in most cases due to the influence of standard curve intercept. Therefore, we recommended that LRG-QAMS method could be used as the substitute of AVG-QAMS method with higher accuracy for quantitative determination only under the condition of zero-tending impact of intercept. Otherwise, the AVG-QAMS method was more suitable and general. 

Interestingly, as for the LRG-QAMS method, the content of the IRS had minimal influence on the SMD values, while a clear numerical reduction trend in the low content region of scatter diagrams was mostly observed when the content of the analytes increased, which coincided with the conclusion mentioned in Wang’s and our previous research [16,17]. We also noticed that the larger the intercept, the more obvious this trend was, even within a relatively wide concentration range. Our previous study has proved that there is no significant influence on the SMDs by changing different component as IRS.

## 4. Materials and Methods

### 4.1. Reagents and Chemicals

**Figure 7:**
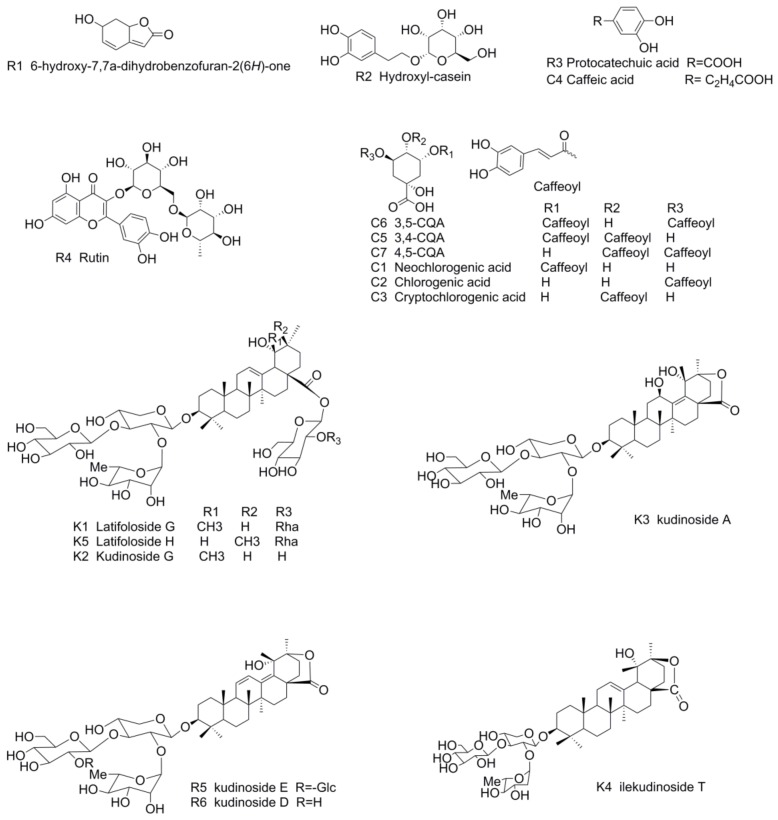
Chemical structures of the 18 target compounds.

Acetonitrile (HPLC grade) was obtained from Merck KGaA (Darmstadt, Germany), and distilled water was purchased from Huaren Yibao Drinks Co. (Hong Kong, China). Phosphoric acid (HPLC grade) was supplied by Tianjin Kemiou Chemical Reagent Co. Ltd. (Tianjin, China). Methanol (A.R.) was bought from Sinopharm Chemical Reagent Co. Ltd. (Shanghai, China). The reference substances of 6-hydroxy-7,7a-ihydrobenzofuran-2(6*H*)-one (R1), hydroxycasein (R2), latifoloside G (K1), kudinoside G (K2), kudinoside A (K3), kudinoside E (R5), kudinside D (R6), ilekudinoside T (K4) and latifoloside H (K5) were isolated from *Ilex kudingcha* C. J. Tseng and their structures were fully characterized by chemical and spectroscopic methods. The other standards, namely protocatechuic acid (3,4-DA, R3), rutin (R4), neochlorogenic acid (5-CQA, C1), chlorogenic acid (3-CQA, C2), cryptochlorogenic acid (4-CQA, C3), caffeic acid (CA, C4), isochlorogenic acid B (3,4-CQA, C5), isochlorogenic acid A (3,5-CQA, C6) and isochlorogenic acid C (4,5-CQA, C7) were purchased from the National Institute for Food and Drug Control (Beijing, China) or Sichuan Weikeqi Biological Technology Co., Ltd. (Qingjiang, Zhonglu, China) Purity analysis proved to be above 98% by HPLC, and their chemical structures are listed in Figure 7.

### 4.2. Plant Materials

Fifteen commercial herbal samples of *Ilex kudingcha* C. J. Tseng (identified with serial numbers 1–15, Table 10) were purchased from Guangzhou drug stores or markets in 2015, and were authenticated by Professor Jin-song Zhou from Guangzhou University of Traditional Chinese Medicine. The voucher specimens were deposited at the Herbarium at Guangzhou University of Traditional Chinese Medicine. The air-dried samples were smashed into powder (40 mesh) and stored in a desiccator.

### 4.3. Instrument and Chromatographic Conditions

Analyses were primarily performed using Waters Alliance E2695 (Waters Corp., Milford, MA, USA) liquid chromatographic system before Shimadzu is indicated comprised of a quaternary solvent delivery system, an online degasser, an auto-sampler and a photodiode array detector (PDA) (Waters Corp.). The separation was performed on Phenomenex Synergi Hydro-RP C_18_ columns (4.6 mm × 250 mm, 5 μm). The mobile phase was composed of 0.05% aqueous phosphoric acid (A, pH 3.0), acetonitrile (B) and methanol (C) using a gradient elution of 6% B at 0–5 min(A-B), 6–12% B at 5–15 min (A-B), 12–16% B at 15–25 min (A-B), 16–20% B and 0–3% C at 25–26 min (A-B-C), 20% B and 3% C at 26–45 min (A-B-C), 20–26% B and 3–0% C at 45–50 min (A-B-C), 26% B at 50–70 min (A-B), 26–29% B at 70–85 min (A-B), 29–46% B at 85–110 min (A-B), 46–90% B at 110–110.01 min (A-B), 90% B at 110.01–120 min (A-B). The flow rate was set at 1.0 mL·min^−1^ with the temperature maintained at 30 °C. The injection volume was 20 μL and the detection wavelength was set at 210 nm, 260 nm and 326 nm simultaneously.

### 4.4. Preparation of Solutions

Approximately 0.2 g of finely ground sample powder (40 mesh) was accurately weighted into a brown stoppered flask and immersed with 20 mL methanol (A.R) at room temperature (25 ± 3 °C) for 30 min, then extracted using ultrasonication in an ice bath for 30 min. After cooling to room temperature, the lost solvent in the extraction solution was replenished with methanol and mixed well. The extract was filtered through a 0.22 μm filter membrane prior to injection.

Appropriate amounts of reference substance of R1, R2, 3,4-DA, 5-CQA, 3-CQA, 4-CQA, CA, R, 3,4-CQA, 3,5-CQA, 4,5-CQA, K1, K2, K3, R5, R6, K4 and K5 were accurately weighed and dissolved in methanol to make the mixed stock solutions with the concentration of 62.0, 380, 10.13, 101.7, 755, 101.0, 106.0, 31.6, 106.6, 1365, 485, 1045, 915, 1065, 60.0, 505, 39.0 and 99.8 μg·mL^−1^ accordingly, then diluted to the appropriate concentration ranges for construction of calibration curves. All solution was stored at 4 °C. The purities of these compounds were all above 98% as determined by HPLC analysis.

### 4.5. Calculation of the Relative Correction Factors (RCFs) and Relative Retention Time (RT_R_)

RCFs of the components co-existing in a TCM were calculated using the standard substance of each analyte by taking a typical active one with characteristic of sufficient abundance in chromatograms and easy availability as the internal reference substance (IRS) according to formula (1) as reported previously. In most QAMS related studies, the final RCF of an analyte is calculated using the average of several RCFs from the IRS and the analyte measured under multiple concentration levels, which would be referred to as average method (AVG-QAMS) in the following passages [12]. In the present study, a novel RCF calculating method, namely linear regression method (LRG-QAMS) was applied based on Equation (3), which used the linear relationship between *C_x_* (i.e., concentration of the analyte) and (*A_x_* × *C_i_*)/*A_i_* (i.e., peak areas of internal referring substance-*A_i_* and the analyte-*A_x_*; concentration of the internal referring substance-*C_i_*) to calculated RCFs by linear regression. The relative retention time was calculated as the ratio of retention time of the analyte versus to that of IRS:

(1) $$RCF_{x}=\frac{f_{x}}{f_{i}}=\frac{A_{x}/C_{x}}{A_{i}/C_{i}}$$

(2) $$C_{x}=\frac{C_{i}}{RCF_{x}}\times \frac{A_{x}}{A_{i}}$$

(3) $$C_{x}\times RCF_{x}=\frac{A_{x}}{\left(A_{i}/C_{i}\right)}$$

where *A_x_* and *C_x_* represent the peak area and concentration of the analyte, respectively. *A_i_* and *C_i_* are the peak area and concentration of the IRS, accordingly.

### 4.6. HCA, PCA and SA Analysis of Samples

The HCA analysis was realized using the SPSS 19.0 software (IBM, Armonk, NY, USA). The between-group linkage method was applied, and euclidean distance was selected as a measurement. Dendrograms resulting from the 18 marker components content were derived from the HPLC profiles of the tested samples.

The PCA analysis was done by the SIMCA-P 12.0 software (Umetrics, Sweden). In this study, content of the detective eighteen markers in 15 batches of samples composed a data matrix with 15 rows and 18 columns, which were used for the PCA analysis after normalization. The first two principal components were extracted, and the scatter plot was obtained by plotting scores of PC1 versus PC2. Euclidean distance, correlation coefficient and cosine were adopted to calculate the similarity of samples as expressed by the following formulae (4)–(6), respectively:

(4) $$d_{ir}={\left[\overset{m}{\underset{k=1}{\sum }}{\left(X_{ik}-X_{rk}\right)}^{2}\right]}^{1/2}$$

(5) $$r_{ir}=\frac{\sum_{k-1}^{m}\left(X_{ik}-{\bar{X}}_{i}\right)\left(X_{rk}-{\bar{X}}_{r}\right)}{\sqrt{\sum_{k-1}^{m}{\left(X_{ik}-{\bar{X}}_{i}\right)}^{2}\sum_{k-1}^{m}{\left(X_{rk}-{\bar{X}}_{r}\right)}^{2}}}$$

(6) $$C_{ir}=\frac{\sum_{k-1}^{m}X_{ik}\cdot X_{rk}}{\sqrt{\left(\sum_{k-1}^{m}X_{ik}^{2}\right)\left(\sum_{k-1}^{m}X_{rk}^{2}\right)}}$$

where *X_ik_* and *X_rk_* represent the *k*th variate of the *i*th sample and mean vector in common pattern, respectively; *X_i_* and *X_r_* are the mean values of them.

## 5. Conclusions

In our study, a validated and sensitive QAMS method with high precision, stability, and repeatability was firstly developed for simultaneous quantification of eighteen analytes in *Ilex kudingcha* C. J. Tseng, as well as to study the influence parameters on the accuracy of the LRG-QAMS and AVG-QAMS method, respectively. Some interesting findings different from previous studies attributed to the diverse results of varied ingredients were illustrated and discussed in our paper. The AVG-QAMS and LRG-QAMS methods could be used as a substitute for the external standard method when standard substances are lacking, and had their respective particulars. Additionally, in order to ensure the high accuracy of the QAMS method, the choice of AVG or LRG was introduced. Moreover, the content of the eighteen analytes from HPLC profiles was applied for HCA, PCA and similarity analysis to identify particular herbs from related species and evaluate the similarities and differences among *Ilex kudingcha* C. J. Tseng samples. This high throughput method has a promising potential to play a very substantial and enhanced role in the development of standards for the quality control of *Ilex kudingcha* C. J. Tseng.

## Figures and Tables

**Table 1:** The results of linearity, relative conversion factors and relative retention time (*n* = 6).

Com.	Standard Curve	R²	Linear Regression Equation of LRG-QAMS	R²	Liner Range	RCF	RT_R_
					(μg/mL)	Mean ± SD	Mean ± SD
R1	y = 118748x − 31340	0.9999	y = 3.169x + 0.480	0.9999	3.10–62.0	3.194 ± 0.088	0.319 ± 0.0002
R2	y = 61195x − 37654	1	y = 1.633x + 3.252	0.9998	19.0–380.0	1.68 ± 0.046	0.359 ± 0.0001
R3	y = 57785x − 2280.2	0.9998	y = 1.542x + 0.0429	1	0.51–10.2	1.57 ± 0.030	0.405 ± 0.0006
C1	y = 39761x + 45253	0.9996	y = 0.790x + 0.899	0.9996	5.09–101.8	0.775 ± 0.025	0.388 ± 0.0003
C2	y = 43436x + 297200	0.9997	y = 0.863x + 5.905	0.9997	37.75–755.0	0.836 ± 0.034	0.541 ± 0.0005
C3	y = 36208x + 43637	0.9992	y = 0.719x + 0.867	0.9992	5.05–101.0	0.709 ± 0.022	0.558 ± 0.0004
C4	y = 90775x + 95028	0.9996	y = 1.803x + 1.888	0.9996	5.30–106.0	1.753 ± 0.062	0.622 ± 0.0006
R4	y = 37462x − 7648.5	0.9998	-	-	1.58–31.6	-	-
C5	y = 86580x + 91283	0.9996	y = 1.720x + 1.814	0.9996	5.33–106.6	1.731 ± 0.057	0.946 ± 0.0003
C6	y = 49744x + 102245	0.9999	-	-	68.25–1365	-	-
C7	y = 66796x + 255530	0.9994	y = 1.327x + 5.077	0.9994	24.25–485.0	1.280 ± 0.045	1.101 ± 0.0003
K1	y = 3028.4x + 11275	1	y = 0.418x + 1.020	1	52.25–1045	0.423 ± 0.005	0.792 ± 0.0010
K2	y = 966.83x + 5437.3	0.9999	y = 0.134x + 0.603	0.9998	45.75–915.0	0.137 ± 0.004	0.899 ± 0.0009
K3	y = 7244.3x + 9510	1	-	-	53.25–1065	-	-
R5	y = 36942x + 4453.1	1	y = 0.986x + 0.540	0.9997	3.00–60.0	1.039 ± 0.039	3.064 ± 0.0018
R6	y = 38462x − 4507.1	1	y = 1.026x + 3.484	0.9998	25.25–505.0	1.066 ± 0.032	3.119 ± 0.0028
K4	y = 4854.9x − 1014.7	0.9999	y = 0.670x − 0.168	1	1.95–39.0	0.635 ± 0.026	1.236 ± 0.0035
K5	y = 2573.6x − 480	1	y = 0.369x − 0.459	1	4.99 ~ 99.8	0.349 ± 0.017	1.244 ± 0.0032

**Table 2:** Precision, repeatability, stability and recovery test results of the HPLC method.

Component	Precision	Repeatability	Stability	Recovery	
	(RSD/%, *n* = 6)	(RSD/%, *n* = 6)	(RSD/%, *n* = 6)	x/%	RSD/%
R1	1.07	1.50	1.60	104.6	5.7
R2	0.74	1.23	0.82	96.9	4.1
R3	2.42	0.66	1.66	98.6	4.9
C1	0.88	2.94	0.87	94.0	2.3
C2	0.61	5.44	0.31	99.6	4.0
C3	1.55	7.05	0.97	96.9	5.2
C4	2.97	7.08	3.39	92.6	3.9
R4	1.82	3.30	1.35	105.3	3.6
C5	1.00	4.40	0.60	102.4	4.7
C6	0.99	4.33	3.10	104.7	3.7
C7	2.45	2.88	1.59	93.4	4.9
K1	0.45	3.91	0.32	100.8	4.6
K2	2.58	6.44	3.30	107.7	5.6
K3	1.05	6.21	1.16	96.7	1.4
R5	3.16	2.61	2.42	95.4	4.8
R6	0.81	4.80	1.22	97.2	3.3
K4	1.37	6.81	1.58	108.5	6.9
K5	2.63	4.05	2.62	105.2	6.6

**Table 3:** Effects of columns and instruments upon RCF and RT_R._

Effects of Column.					Effects of Instrument			
	RCF		RT_R_		RCF		RT_R_	
	Mean	RSD%	Mean	RSD%	Mean	RSD%	Mean	RSD%
R1/R4	3.313	0.99	0.316	4.66	3.294	4.01	0.334	4.49
R2/R4	1.743	2.33	0.368	3.77	1.695	2.65	0.402	4.87
R3/R4	1.552	4.37	0.404	3.97	1.543	1.66	0.445	4.63
C1/C6	0.77	4.55	0.389	3.35	0.765	4.33	0.409	4.55
C2/C6	0.827	3.88	0.536	2.96	0.813	4.57	0.566	4.08
C3/C6	0.735	4.58	0.565	1.24	0.742	5.17	0.584	4.29
C4/C6	1.714	1.61	0.616	2.91	1.732	4.68	0.642	3.89
R4	1	0	1	0	1	0	1	0
C5/C6	1.681	1.75	0.955	1.61	1.754	2.31	0.941	0.34
C6	1	0	1	0	1	0	1	0
C7/C6	1.318	4.7	1.103	0.6	1.388	1.32	1.108	2.23
K1/K3	0.421	3.91	0.811	3.62	0.42	4.33	0.794	4.29
K2/K3	0.151	1.67	0.916	2.41	0.143	4.75	0.908	4.92
K3	1	0	1	0	1	0	1	0
R5/R4	1.077	3.82	3.12	4.94	1.081	2.06	3.044	3.55
R6/R4	1.112	1.45	3.171	4.68	1.085	2.83	3.093	3.58
K4/K3	0.662	4.82	1.235	3.09	0.647	4.02	1.181	4.22
K5/K3	0.341	4.86	1.242	2.67	0.352	1.56	1.189	4.19

**Table 4:** Effects of flow rate and column temperature upon RCF and RT_R_.

Effects of Flow Rate					Effects of Column Temperature			
	RCF		RT_R_		RCF		RT_R_	
	Mean	RSD%	Mean	RSD%	Mean	RSD%	Mean	RSD%
R1/R4	3.351	2.8	0.326	2.32	3.36	4.24	0.344	4.36
R2/R4	1.718	1.45	0.367	2.06	1.725	1.64	0.383	4.58
R3/R4	1.569	1.6	0.418	3.4	1.581	4.36	0.429	4.59
C1/C6	0.731	1.82	0.394	1.28	0.737	4.14	0.398	2.64
C2/C6	0.811	2.69	0.543	0.28	0.807	4.62	0.546	0.94
C3/C6	0.678	3.76	0.56	0.47	0.705	1.14	0.561	0.67
C4/C6	1.736	3.17	0.628	0.97	1.735	2.74	0.63	1.39
R4	1	0	1	0	1	0	1	0
C5/C6	1.673	2.19	0.942	0.48	1.661	4.29	0.94	0.54
C6	1	0	1	0	1	0	1	0
C7/C6	1.304	5.31	1.105	0.36	1.31	3.61	1.114	1.05
K1/K3	0.428	3.11	0.787	0.51	0.417	3	0.805	1.59
K2/K3	0.144	4.48	0.894	0.5	0.146	3.44	0.91	1.04
K3	1	0	1	0	1	0	1	0
R5/R4	1.04	4.04	3.03	1.17	1.076	3.07	3.01	1.61
R6/R4	1.086	2.03	3.083	1.23	1.054	4.95	3.062	1.69
K4/K3	0.681	4.92	1.238	0.12	0.617	5.03	1.201	2.77
K5/K3	0.37	2.91	1.248	0.09	0.338	6.24	1.208	2.8

**Table 5:** The content results of the 18 target compounds calculated by external standard method (mg/mL).

Comp.	1	2	3	4	5	6	7	8	9	10	11	12	13	14	15
R1	1.75	0.39	0.58	1.05	0.40	0.24	0.71	0.49	2.06	2.25	0.74	1.41	0.84	0.24	1.42
R2	32.40	4.72	3.42	9.08	2.54	1.60	5.03	2.75	1.08	10.98	6.48	9.52	8.40	1.66	6.42
R3	0.15	0.15	0.48	0.21	0.44	0.31	0.26	0.18	0.57	0.56	0.33	0.21	0.30	0.29	0.24
C1	3.79	5.99	3.91	4.81	4.35	3.43	2.67	4.22	1.92	1.66	3.46	4.38	2.89	4.28	6.47
C2	16.31	25.77	14.05	18.67	13.16	11.60	12.30	16.17	6.82	6.22	16.06	17.49	10.68	15.87	22.85
C3	1.00	5.06	4.62	4.00	5.12	3.73	3.55	4.57	2.03	1.83	4.65	4.42	3.75	4.73	5.62
C4	2.10	0.72	1.51	0.90	1.41	1.13	1.02	0.54	0.57	0.55	3.07	0.48	1.77	1.49	1.02
R4	0.64	0.86	0.43	0.61	0.37	0.37	0.41	0.30	0.26	0.23	1.01	1.54	0.65	0.39	1.29
C5	1.47	3.56	4.19	3.87	4.71	3.48	3.62	3.90	4.02	3.50	4.56	4.47	4.66	4.87	4.76
C6	152.9	45.02	30.61	50.25	39.13	28.22	33.52	33.51	68.65	65.94	38.06	65.10	35.10	43.69	57.06
C7	19.01	12.89	13.19	14.97	16.61	11.07	12.11	9.85	19.39	19.62	21.31	23.32	20.79	20.33	21.28
K1	27.86	13.50	7.96	13.01	9.01	6.32	15.26	9.52	19.38	18.28	25.89	34.59	22.91	15.56	19.94
K2	32.72	10.02	6.35	11.21	5.99	3.71	11.53	4.20	14.43	13.51	26.47	31.47	22.27	10.79	14.77
K3	29.94	25.74	24.73	21.45	24.68	20.90	22.49	21.24	24.55	24.58	34.87	38.46	25.37	34.07	29.78
R5	0.27	0.33	0.32	0.38	0.45	0.36	0.40	0.39	3.41	0.30	0.75	0.93	0.81	0.60	0.52
R6	3.76	3.93	3.99	2.92	3.74	2.95	3.06	2.80	3.31	3.01	5.44	5.74	4.88	5.45	3.81
K4	0.29	0.28	0.23	0.11	0.45	0.25	0.13	0.34	0.27	0.27	0.84	0.78	0.65	0.48	0.46
K5	0.64	0.58	0.78	0.55	0.81	0.57	0.64	0.48	0.68	0.51	0.37	0.35	0.31	0.98	0.63

**Table 6:** The content of the marker components detected at 326 nm using both LRG-QAMS and AVG-QAMS method for data processing (mg/g).

Location		1	2	3	4	5	6	7	8	9	10	11	12	13	14	15
LRG-QAMS	C1	3.83	6.04	3.94	4.85	4.38	3.45	2.69	4.24	1.93	1.68	3.48	4.42	2.91	4.32	6.53
	SMD	1.09%	0.75%	0.53%	0.80%	0.68%	0.48%	0.60%	0.59%	0.94%	0.94%	0.67%	0.90%	0.63%	0.74%	0.84%
	C2	16.50	25.99	14.14	18.83	13.26	11.66	12.38	16.26	6.88	6.28	16.17	17.65	10.75	15.99	23.05
	SMD	1.10%	0.75%	0.54%	0.81%	0.70%	0.49%	0.61%	0.60%	0.98%	0.97%	0.68%	0.91%	0.64%	0.75%	0.85%
	C3	1.01	5.10	4.65	4.04	5.16	3.74	3.58	4.59	2.05	1.85	4.68	4.46	3.77	4.76	5.67
	SMD	1.19%	0.75%	0.53%	0.80%	0.68%	0.48%	0.60%	0.59%	0.94%	0.94%	0.67%	0.90%	0.62%	0.74%	0.85%
	C4	2.11	0.67	1.56	0.87	1.46	1.13	1.01	0.46	0.50	0.47	3.07	0.41	1.78	1.50	1.03
	SMD	0.66%	−7.03%	3.30%	−3.00%	2.83%	−0.01%	−1.29%	−14.1%	−12.4%	−13.5%	−0.01%	−14.3%	0.34%	0.64%	1.27%
	C5	1.49	3.59	4.21	3.90	4.74	3.50	3.65	3.92	4.06	3.54	4.59	4.51	4.68	4.91	4.80
	SMD	1.13%	0.75%	0.52%	0.80%	0.67%	0.47%	0.59%	0.59%	0.91%	0.90%	0.66%	0.89%	0.61%	0.73%	0.84%
	C7	19.21	12.98	13.26	15.09	16.72	11.13	12.18	9.91	19.56	19.80	21.46	23.53	20.92	20.48	21.46
	SMD	1.08%	0.75%	0.53%	0.80%	0.68%	0.47%	0.59%	0.60%	0.91%	0.89%	0.66%	0.89%	0.61%	0.73%	0.84%
AVG-QAMS	C1	3.70	5.76	3.79	4.64	4.20	3.33	2.62	4.08	1.92	1.68	3.37	4.25	2.83	4.15	6.22
	SMD	−2.52%	−3.88%	−3.13%	−3.39%	−3.26%	−2.80%	−1.80%	−3.27%	0.09%	0.93%	−2.65%	−3.08%	−2.07%	−3.18%	−3.92%
	C2	16.29	25.29	14.05	18.51	13.22	11.71	12.39	16.07	7.17	6.60	15.98	17.38	10.84	15.81	22.50
	SMD	−0.15%	−1.94%	−0.04%	−0.93%	0.42%	0.88%	0.68%	−0.59%	5.28%	6.19%	−0.49%	−0.60%	1.52%	−0.37%	−1.52%
	C3	1.06	4.90	4.48	3.90	4.95	3.63	3.47	4.43	2.04	1.85	4.51	4.30	3.65	4.58	5.44
	SMD	6.31%	−3.18%	−3.17%	−2.54%	−3.27%	−2.63%	−2.37%	−3.09%	0.35%	0.94%	−3.06%	−2.72%	−2.51%	−3.03%	−3.31%
	C4	2.10	0.73	1.58	0.92	1.48	1.16	1.05	0.53	0.56	0.54	3.02	0.48	1.78	1.52	1.07
	SMD	0.28%	0.54%	4.36%	2.11%	4.27%	3.06%	2.64%	−2.25%	−1.42%	−1.84%	−1.63%	−0.70%	0.71%	1.89%	5.11%
	C5	1.51	3.49	4.08	3.79	4.59	3.41	3.55	3.81	3.93	3.44	4.44	4.36	4.53	4.74	4.64
	SMD	2.38%	−1.94%	−2.58%	−2.12%	−2.70%	−2.14%	−2.14%	−2.34%	−2.11%	−1.76%	−2.64%	−2.38%	−2.73%	−2.72%	−2.56%
	C7	18.98	12.94	13.21	14.98	16.56	11.14	12.17	9.97	19.31	19.54	21.15	23.15	20.63	20.20	21.15
	SMD	−0.18%	0.43%	0.15%	0.08%	−0.28%	0.63%	0.46%	1.17%	−0.38%	−0.42%	−0.80%	−0.72%	−0.80%	−0.64%	−0.61%

**Table 7:** The content of the marker components detected at 260 nm using both LRG-QAMS and AVG-QAMS method for data processing (mg/g).

Location		1	2	3	4	5	6	7	8	9	10	11	12	13	14	15
LRG-QAMS	R1	1.76	0.36	0.57	1.04	0.38	0.23	0.70	0.48	2.20	2.43	0.72	1.39	0.83	0.23	1.40
	SMD	0.89%	−8.28%	−2.37%	−0.60%	−4.90%	−2.78%	−0.87%	−1.51%	6.58%	8.01%	−3.59%	−1.60%	−1.78%	−2.95%	−1.33%
	R2	33.21	4.57	3.33	9.13	2.42	1.54	5.03	2.69	1.00	11.81	6.35	9.39	8.41	1.60	6.27
	SMD	2.51%	−3.11%	−2.73%	0.58%	−4.56%	−3.91%	−0.03%	−2.21%	−7.01%	7.55%	−1.95%	−1.37%	0.14%	−3.68%	−2.44%
	R3	0.15	0.15	0.49	0.21	0.45	0.32	0.27	0.19	0.62	0.61	0.33	0.20	0.30	0.30	0.23
	SMD	−1.18%	−1.98%	3.51%	0.23%	4.24%	3.55%	2.53%	3.60%	7.47%	8.70%	0.03%	−1.94%	0.97%	3.21%	−1.27%
	R5	0.24	0.30	0.30	0.36	0.44	0.35	0.39	0.38	3.66	0.29	0.73	0.90	0.79	0.59	0.50
	SMD	−11.87%	−9.15%	−6.97%	−5.96%	−1.24%	−4.21%	−3.32%	−1.47%	7.51%	−2.94%	−3.51%	−3.19%	−1.97%	−1.35%	−3.90%
	R6	3.53	3.67	3.84	2.67	3.60	2.77	2.87	2.65	3.25	2.96	5.20	5.47	4.69	5.40	3.53
	SMD	−6.03%	−6.50%	−3.84%	−8.56%	−3.57%	−6.11%	−6.28%	−5.14%	−1.91%	−1.73%	−4.36%	−4.75%	−3.94%	−0.83%	−7.57%
AVG-QAMS	R1	1.77	0.37	0.58	1.05	0.39	0.25	0.71	0.49	2.20	2.43	0.73	1.40	0.84	0.25	1.41
	SMD	0.96%	−5.17%	−0.55%	0.07%	−1.89%	2.71%	0.49%	0.82%	6.49%	7.84%	−2.31%	−1.30%	−0.76%	2.52%	−1.04%
	R2	32.49	4.64	3.43	9.07	2.55	1.69	5.08	2.81	1.17	11.67	6.37	9.33	8.37	1.75	6.29
	SMD	0.26%	−1.69%	0.23%	−0.08%	0.41%	5.50%	1.04%	2.12%	8.40%	6.33%	−1.68%	−2.08%	−0.34%	5.31%	−2.13%
	R3	0.15	0.15	0.49	0.21	0.45	0.32	0.26	0.19	0.61	0.61	0.33	0.20	0.30	0.30	0.23
	SMD	−1.11%	−1.92%	2.29%	−0.23%	3.05%	2.63%	1.80%	3.29%	6.08%	7.30%	−0.88%	−2.31%	0.13%	2.36%	−1.82%
	R5	0.28	0.34	0.34	0.39	0.47	0.38	0.42	0.41	3.53	0.33	0.74	0.90	0.80	0.61	0.53
	SMD	2.73%	1.87%	4.38%	2.89%	5.24%	5.21%	4.61%	6.84%	3.48%	9.36%	−1.61%	−2.60%	−0.59%	2.19%	1.06%
	R6	3.73	3.86	4.02	2.90	3.80	2.99	3.09	2.88	3.46	3.17	5.33	5.59	4.84	5.53	3.72
	SMD	−0.83%	−1.65%	0.78%	−0.77%	1.60%	1.48%	0.92%	3.02%	4.31%	5.49%	−1.91%	−2.59%	−0.81%	1.48%	−2.44%

**Table 8:** The content of the marker components detected at 210 nm using both LRG-QAMS and AVG-QAMS method for data processing (mg/g).

Location		1	2	3	4	5	6	7	8	9	10	11	12	13	14	15
LRG-QAMS	K1	27.93	13.62	8.11	13.12	9.15	6.47	15.36	9.65	19.47	18.38	25.99	34.67	22.98	15.69	20.04
	SMD	0.26%	0.90%	1.83%	0.85%	1.56%	2.33%	0.66%	1.36%	0.46%	0.51%	0.37%	0.23%	0.33%	0.84%	0.52%
	K2	32.68	10.08	6.42	11.25	6.08	3.80	11.58	4.28	14.46	13.55	26.48	31.48	22.26	10.86	14.81
	SMD	−0.14%	0.54%	1.13%	0.32%	1.23%	2.23%	0.32%	1.90%	0.18%	0.23%	0.00%	−0.03%	−0.06%	0.59%	0.26%
	K4	0.31	0.30	0.24	0.13	0.47	0.27	0.15	0.35	0.28	0.29	0.84	0.78	0.66	0.49	0.47
	SMD	5.53%	5.72%	7.43%	16.25%	2.96%	6.36%	14.40%	4.39%	6.05%	5.94%	0.52%	0.77%	1.22%	2.76%	2.89%
	K5	0.68	0.62	0.82	0.59	0.84	0.61	0.68	0.53	0.72	0.54	0.42	0.40	0.36	1.01	0.67
	SMD	6.21%	7.00%	4.43%	7.47%	4.19%	7.13%	6.03%	8.87%	5.49%	8.38%	12.68%	13.51%	15.61%	3.11%	6.36%
AVG-QAMS	K1	27.76	13.63	8.19	13.13	9.22	6.57	15.35	9.71	19.40	18.32	25.84	34.41	22.87	15.68	19.97
	SMD	−0.36%	0.97%	2.83%	0.97%	2.29%	3.92%	0.58%	1.99%	0.12%	0.24%	−0.20%	−0.52%	−0.15%	0.73%	0.16%
	K2	32.33	10.28	6.70	11.42	6.36	4.04	11.73	4.61	14.55	13.66	26.27	31.15	22.16	11.03	14.89
	SMD	−1.18%	2.52%	5.66%	1.84%	6.16%	8.97%	1.74%	9.97%	0.84%	1.09%	−0.73%	−1.02%	−0.48%	2.26%	0.84%
	K4	0.30	0.29	0.23	0.12	0.47	0.26	0.13	0.35	0.27	0.28	0.86	0.80	0.67	0.49	0.47
	SMD	2.83%	2.76%	2.74%	2.65%	2.73%	2.64%	2.68%	2.65%	2.73%	2.73%	2.89%	2.93%	2.75%	2.88%	2.83%
	K5	0.66	0.60	0.82	0.57	0.85	0.59	0.67	0.50	0.71	0.52	0.38	0.36	0.31	1.04	0.65
	SMD	4.28%	3.91%	4.78%	3.60%	4.87%	3.70%	4.15%	3.10%	4.41%	3.39%	2.09%	1.84%	0.87%	5.45%	4.23%

**Table 9:** Similarity analysis results of 15 samples from different habitats.

Sample No.	Euclidean Distance (4)	Correlation (5)	Cosine (6)
1	0.900	0.949	0.950
2	0.988	0.973	0.978
3	0.980	0.980	0.982
4	0.981	0.995	0.937
5	0.987	0.985	0.988
6	0.977	0.984	0.986
7	0.982	0.996	0.996
8	0.981	0.982	0.985
9	0.980	0.976	0.977
10	0.983	0.983	0.985
11	0.985	0.963	0.968
12	0.983	0.997	0.998
13	0.965	0.953	0.960
14	0.989	0.977	0.981
15	0.989	0.993	0.995

Remark: these data calculated by formula (4)–(6).

**Table 10:** The collection and descriptions of the tested samples.

Num.	Province/City	Date of Purchase
1	Hainan/Haikou	6 June 2015
2	Hunan/Bozhou	12 June 2015
3	Guangxi/Daxin	30 May 2015
4	Anhui/Huangshan	19 June 2015
5	Sichuan/Chengdu	2 May 2015
6	Guangxi/Nanning	30 May 2015
7	Shanghai	26 May 2015
8	Yunnan/Shaotong	10 June 2015
9	Guangdong/Foshan	20 May 2015
10	Guangdong/Guangzhou	23 May 2015
11	Sichuan/Mianyang	2 June 2015
12	Guangdong/Zhongshan	23 May 2015
13	Hunan/Xiangtan	12 June 2015
14	Jiangxi/Jinggangshan	9 June 2015
15	Guangdong/Zhanjiang	26 May 2015

## Abbreviations

(QAMS)	quantitative analysis of multi-components with single marker
(IRS)	internal referring substances
(RCFs)	relative correction factors
(LRG)	linear regression
(AVG)	average method
(TCM)	traditional Chinese medicine
(HCA)	hierarchical clustering analysis
(PCA)	principal components analysis
(SA)	similarity analysis
(TCM)	traditional Chinese medicine
(ES)	external standard
(PDA)	relative retention time
(RTR)	relative retention time
(RSD)	relative standard deviation
